# Patient-Centered Communication Case: Threatened Miscarriage

**DOI:** 10.21980/J8.52360

**Published:** 2025-12-31

**Authors:** Anita Rohra, Daniela Ortiz, Shagun Berry, Colleen Donovan, Nicole Novotny, Stephanie Cohen, Charles Lei, Alaa Aldalati, Stephanie Stapleton, David Fernandez

**Affiliations:** 1 Baylor College of Medicine, Department of Emergency Medicine, Houston, TX; 2 Rush University Medical Center, Department of Emergency Medicine, Chicago, IL; 3 Rutgers Robert Wood Johnson Medical School, Department of Emergency Medicine, New Brunswick, NJ; 4 Ochnser Health System, Department of Emergency Medicine, New Orleans, LA; 5 University of Central Florida, Department of Emergency Medicine, Orlando, FL; 6 Hennepin County Medical Center, Department of Emergency Medicine, Minneapolis, MN; 7 University of Kansas School of Medicine-Wichita, Wesley Medical Center Emergency Department, Wichita, KS; 8 Boston University/Boston Medical Center, Department of Emergency Medicine, Boston, MA; 9 Mount Sinai Hospital, Department of Emergency Medicine, Brooklyn, NY

## Abstract

**Audience:**

This communication case is intended for EM residents of all levels.

**Introduction:**

Patient-centered communication is a necessary skill in the practice of emergency medicine. This style of communication is crucial for promoting high-quality healthcare by prioritizing patient needs, perspectives, and values. This patient-centered communication case centers on miscarriage, a diagnosis where patient-centered communication is requisite. Approximately one in six or 17.1% of patients with a final diagnosis of miscarriage, also known as early pregnancy loss, present initially to the emergency department.^1^ Advising a patient of the diagnosis of miscarriage requires excellent communication skills including facile rapport building, empathy, nonverbal communication, and explanation of management options.

**Educational Objectives:**

By the end of this certifying exam practice case, learners will be able to: 1) establish a supportive and compassionate environment through verbal and non-verbal communication when engaging with a patient experiencing distress, anxiety, or grief related to potential pregnancy loss, 2) actively explore the patient’s understanding, concerns, values, and goals related to their pregnancy and presenting symptoms, 3) recognize and normalize a range of emotional reactions, offering validation and support regardless of the patient’s obstetric history or desired pregnancy outcomes, 4) clearly explain the diagnosis of a “threatened miscarriage,” outlining its clinical implications, inherent uncertainty, and potential outcomes, 5) review the results of any imaging or lab studies succinctly and empathetically, while verifying the patient’s understanding, 6) collaborate with the patient to develop a mutually agreeable care plan, including medical recommendations, appropriate follow-up, monitoring, and return precautions.

**Educational Methods:**

This standardized patient case provides an opportunity to practice patient-centered communication and debrief on areas for improvement for the learner. The case was co-developed by experts in simulation-based education and emergency medicine resident leadership.

**Research Methods:**

Facilitators evaluated the standardized patient case via a survey for efficacy, while learners evaluated it via a survey from the learner perspective. This case was tested in a serial fashion with incremental improvements based on feedback at each step: initially, both learners and facilitators at the case writer’s institution, then learners and facilitators at the annual SAEM meeting, and finally with both at an institution outside of the case writer’s institution. Three unique learners and three unique facilitators tested the case throughout the entire process.

**Results:**

The case was reviewed favorably with minor recommendations noted, such as additional notes for the facilitator and stronger alignment between objectives and critical actions expected. Both surveys asked for demographic information and an evaluation of the case on a scale of 1–5, with 5 being the highest rating. Learners ranked the case 4.5, and then 4.8 on iterative trialing sessions. Facilitators ranked the case above 4 out of 5 for all questions. Comments centered on clarifying verbal prompts and debriefing plan.

**Discussion:**

Overall, this standardized patient case for patient communication was received positively and is recommended for use in preparation for the ABEM certifying examination.

**Topics:**

Patient-centered communication, threatened miscarriage, health communication.

## USER GUIDE

**Table:** 

List of Resources:	
Abstract	58
User Guide	60
For Examiner Only	62
Certifying Exam Assessment	67
Stimulus	69
Debriefing and Evaluation Pearls	74

**Learner Audience:**

EM residents of all levels

**Time Required for Implementation:**

Case: 10 minutes

Debriefing: 5–10 minutes

**Recommended number of learners per instructor:**

one

**Topics:**

Patient-centered care, threatened abortion, health communication.

**Objectives:**

By the end of this certifying exam practice case, learners will be able to:

1. Establish a supportive and compassionate environment through verbal and non-verbal communication when engaging with a patient experiencing distress, anxiety, or grief related to potential pregnancy loss.
2. Actively explore the patient’s understanding, concerns, values, and goals related to their pregnancy and presenting symptoms.
3. Recognize and normalize a range of emotional reactions, offering validation and support regardless of the patient’s obstetric history or desired pregnancy outcomes.
4. Clearly explain the diagnosis of a “threatened miscarriage,” outlining its clinical implications, inherent uncertainty, and potential outcomes.
5. Review the results of any imaging or lab studies succinctly and empathetically.
6. Collaborate with the patient to develop a mutually agreeable care plan, including medical recommendations, appropriate follow-up, monitoring, and return precautions.
7. Identify shared goals in optimizing patient care (reducing myocardial damage and preventing deterioration) and use these to negotiate a mutually acceptable plan.

### Linked objectives, methods and results

This standardized patient (SP) case involves a 28-year-old female presenting with vaginal bleeding and cramping at seven weeks gestation. The learner is provided with minimal initial data from the candidate task sheet prior to starting the patient encounter. The learner is expected to explore the patient’s understanding of the situation, including what they believe is happening and how they feel about the pregnancy. (Objectives 1 and 2) As the patient shares her fears and hopes, the learner must demonstrate empathy and nonjudgmental support for the patient’s emotional response, whether grief, uncertainty, or guarded optimism. (Objective 3) The learner will receive the results of the patient’s physical exam and diagnostic workup, including an ultrasound report showing an intrauterine pregnancy with a mild subchorionic hemorrhage, and lab work without other critical findings. Based on this, the learner must clearly explain the diagnosis of a threatened miscarriage and communicate uncertainty in a balanced and informative way. (Objectives 4 and 5) The learner should then work with the patient to develop a care plan that covers follow-up options, return precautions, and symptom monitoring, while tailoring the conversation to the patient’s emotional and informational needs. The session concludes with the learner checking for understanding, addressing any final concerns, and summarizing next steps while maintaining an empathetic tone and professional presence throughout. (Objective 6)

**Topics:**

### Recommended pre-reading for instructor

- Heaton HA. Ectopic pregnancy and emergencies in the first 20 weeks of pregnancy. In: Tintinalli JE, Stapczynski J, Ma O, Yealy DM, Meckler GD, Cline DM, eds. *Tintinalli’s Emergency Medicine: A Comprehensive Study Guide.* 8^th^ ed. New York: McGraw-Hill Education; 2016.
- Salhi BA, Nagrani S. Acute complications of pregnancy. In: Walls RM, Hockberger RS, Gausche-Hill M, et al, eds. *Rosen’s Emergency Medicine: Concepts and Clinical Practice*. 9^th^ ed. Philadelphia, PA: Elsevier; 2018.
- Hashim MJ. Patient-centered communication: Basic skills. *Am Fam Physician*. 2017;95(1):29–34.

### Results and tips for successful implementation

This case can be used during a mock certifying exam practice session, along with other cases, or in isolation. For the most authentic practice, we recommend using a ratio of one faculty to one resident. When needed, multiple learners may also practice together in small groups. Residents may use a mock facilitator role to better understand an examiner’s perspective. The case should be completed within 15 minutes, and feedback given within five minutes.

We used an iterative case-trial process across multiple sites with a convenience sample of EM residents. Both the facilitators and residents provided feedback on their experiences via anonymous surveys with Likert scales and open comment sections. Likert scales ranged from 1 (strongly disagree) to 5 (strongly agree). All data were collected using Qualtrics (https://www.qualtrics.com) and analyzed using Excel (Microsoft, Redmond, WA). The Boston University Institutional Review Board reviewed the project and deemed it exempt.

After the written review, facilitators completed an SSET survey 2 to evaluate the quality of key simulation elements. During the first round of trialing, a facilitator at an alternate academic site tested the case with EM residents. Facilitators completed an SSET survey,^2^ and residents completed a modified usability survey . We performed a second round of case trialing at the Society for Academic Emergency Medicine Annual Meeting during May 2025 (Philadelphia, PA). Mock examiners and examinees completed modified usability surveys. We modified the case after each review by incorporating survey feedback.

We obtained case feedback from a total of five learners and two facilitators over three iterative reviews. The written review (n=1) was largely positive; case objectives, key actions, and materials were clear, but review identified need for clearer prompts and debriefing plan. The first trial users (N=2) ranked the usability as 4.5. The second trial received generally positive feedback. The mock examiner (n=1) found the case easy to use, thought others would feel similarly, and would like to use this case for ABEM certifying exam practice. An area for improvement noted by the facilitator group was to add additional instructions for the facilitator. All residents (n=3 ) ranked the usability at 4.8, finding the written and verbal case materials to be clear, and it was helpful practice for the ABEM certifying exam. Comments for improvement included adding additional lab values that would be ordered in the general care of this type of patient.

## Figures and Tables

**Table:** KEY DECISION POINTS and REQUIRED LEARNER ACTIONS

Decision Point	SP Prompt/Line	Required Learner Action
Recognition of emotional distress	“I’m worried.”	Acknowledge and validate emotions
Clarifying results	“Can you explain the results to me more plainly?”	Avoid jargon, provide clear explanation
Emotional guilt/conflict	“Did I do something wrong to bring this on?”	Normalize experience, offer reassurance
Clinical uncertainty	“How certain are you about the results?”	Acknowledge uncertainty, provide context
Future planning	“What are my options?”	Provide options, elicit patient goals
Safety concerns	“What if the pain and bleeding don’t improve?”	Provide return precautions

**Table:** 

**Candidate Task Sheet**	
**#1**	**Physical Exam Findings**
**#2**	**Laboratory Results**
**#3**	**Pelvic Ultrasound Results**

**Table:** Patient-Centered Communication Candidate Task Sheet

**CASE PARAMETERS**	
This is a 10-minute case. You will interact with a patient and a nurse colleague. You will **not** perform a physical exam. You will discuss the chief complaint, history, differential diagnosis, and management plan with the patient. You will answer the patient’s questions and explain next steps.	
**PATIENT INFORMATION**	
**Patient Name**	Sofia Gonzalez
**Age**	28
**Gender Identity**	Female
**Presenting Complaint**	Vaginal bleeding and abdominal cramping
**General Appearance/His-tory of Present Illness**	Appears uncomfortable, tearful, and anxious; last menstrual period seven weeks ago; reports onset of vaginal spotting with blood and intermittent episodes of lower abdominal cramping since yesterday evening
**Past Medical History**	None
**Medication**	None
**Allergies**	No known allergies
**Vital Signs on ED Arrival**	BP 116/72, HR 88, SpO2 100% on room air, Temp 37°C, RR 16/min
**RESULTS/FINDINGS**	
You will not be performing the physical exam. You will be provided with the results of the physical exam, laboratory studies, and imaging to discuss with the patient.	
**TASK STATEMENT**	
Your task is to meet with Sofia and engage in a patient-centered encounter regarding the reason(s) for her visit and to conclude the case with a clearly communicated plan of care.	

**Stimulus 1 t5-jetem-10-5-ce58:** Physical Exam Findings

Physical Examination	
**GENERAL APPEARANCE**	No apparent distress
**DERMATOLOGIC**	Normal
**HEENT**	Normal
**RESPIRATORY**	Lungs clear to auscultation bilaterally
**CARDIAC**	Normal heart sounds
**ABDOMINAL**	Mild lower abdominal tenderness
**GENITOURINARY**	Vaginal spotting; no cervical motion tenderness on bimanual exam, cervical os closed
**EXTREMITIES**	Full strength, no deformities
**NEUROLOGIC**	No gross motor or sensory deficits

**Stimulus 2 t6-jetem-10-5-ce58:** Laboratory Results

Type	Units	Normal Values
**Lab Results**		
CBC Result	Hemoglobin 12 g/dL Hematocrit 36% White blood cells 12,000/mm3 Platelets 200 x 10^3/mm3	Hemoglobin 12–16 g/dL (female) Hematocrit 36–47% (female) White blood cells 3,200–9,800/mm3 Thrombocytes (platelets) 150–450 x 10^3/mm3
Pregnancy Test Result	Positive	N/A
Rh Type Results	AB+	N/A

**Stimulus 3 f1-jetem-10-5-ce58:**
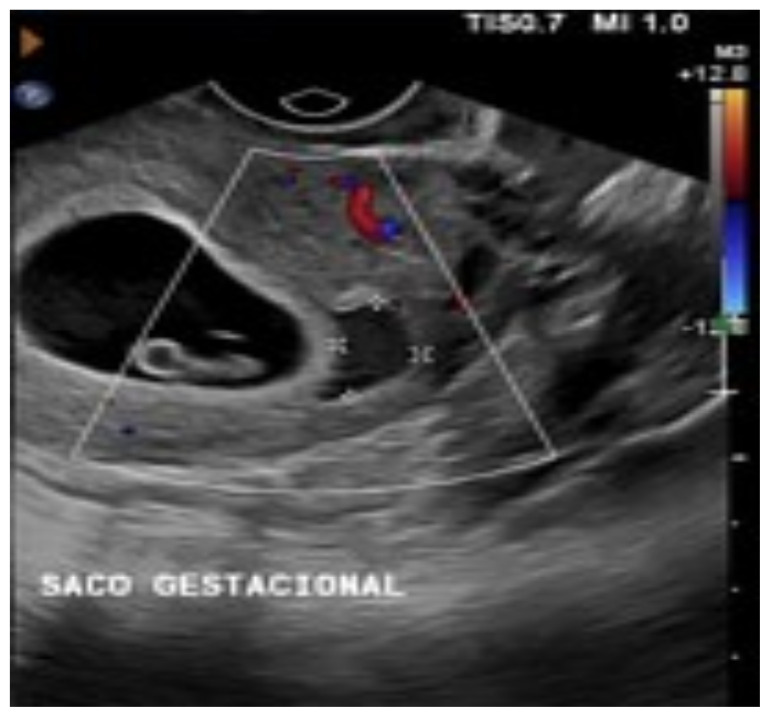
Pelvic Ultrasound Results^9^ Gestational sac with yolk sac and early amnion visible on pelvic ultrasound; mild subchorionic hemorrhage noted. FHR on additional images noted to be 120.

**Table:** Patient Centered Communication Guidance

*Chronological Approach*	*Explicit examples, questions, and statements*
1. Introduce yourself and build rapport	Fully introduce all parties in the room and ensure comfort for all (family, friends, colleagues)
2. Elicit the reason for the patient’s visit with you	“What brings you to the ED today?” or “How can I help you?” to focus the patient’s concern(s).
3. Summarize the patient’s concerns to ensure understanding	Continue to elicit concerns until the patient advises there are no more. “What else?”
4. Prioritize the agenda with the patient to ensure the highest priority items are addressed	“Which of these do you definitively want addressed today?” “What is most concerning to you?”
5. Using open ended questions, obtain a full history of present illness regarding the highest priority concerns	“Tell me more about...” “What was . . . like?”
6. Ask more direct question to obtain full details and perform an ROS	Use the standard expansion of chief complaints details such as duration, severity, location, etc, to elicit details
7. Elicit the patient’s perspective	“What are your thoughts on what we have discussed so far?”
8. Empathize with their concern and perpsective	“Thank you for discussing everything with me and I understand that this is a frustrating experience for you.” “I see that you are worried, and I will do my best to get you the answers you are looking for.”
9. Summarize the full concern(s).	“I would like to summarize and confirm I have everything important that you noted.” “Let’s put everything together and ensure we have all the details.”
10. Transition to obtaining data outside of the patient’s concern including PMH, Family hx, Social hx, social support and resources available to the patient.	“I need to ask some additional questions that may seem unrelated, but we won’t know unless we discuss them.” “I would like about your medical history outside of today’s concern to ensure nothing else is contributory.”

Modified from **Hashim, M.J.** Patient-Centered Communication: Basic skills – Table 1. In: Hashim, M. J. *Patient-Centered Communication*: *Basic Skills* [electronic resource]. Houston, TX: American Academy of Family Physicians; 2017. © American Academy of Family Physicians. https://www.aafp.org/pubs/afp/issues/2017/0101/p29.html

## FOR EXAMINER ONLY

### Patient-Centered Communication Case: Threatened Miscarriage Summary

**Diagnosis:** Threatened miscarriage

**Summary of patient-centered communication**: The learner should discuss the diagnosis of miscarriage with the patient using clear language and ensure the patient understands the diagnosis. The learner should then empathize with the patient and answer any questions the patient has on causes of miscarriage, prevention of miscarriage, and next steps once miscarriage is diagnosed. Once the patient has absorbed the diagnosis and information provided, the learner should elicit the patient’s perspective and use shared decision-making to plan next steps, including return precautions to the emergency department.

**Standardized Actor Profile:**

20–30 yo female, healthy appearing, average health literacy, in a safe relationship with a long-term male partner, average income, and manages a small business, no religious affiliation. The patient has a friend who has had a miscarriage, so she has some experience with this in her personal life. The patient also has a sister who had a complication due to pregnancy and is now having a harder time getting pregnant. This is her first pregnancy, and it is desired.

**Materials/personnel needed:**

Standardized patient (SP) as patient, standardized patient as nurse colleague, stimuli with physical exam findings, laboratory results, and ultrasound images with written report.

**Room Setup:**

1. Exam table with SP seated with a gown
2. Nurse in the room holding physical exam and diagnostic results sheets
3. Chair for learner/examinee

**Play of Case***:* A 28-year-old female with her last menstrual period seven weeks ago and no significant past medical history presents to the emergency department with vaginal bleeding and lower abdominal cramping for two to three days. The examinee is to actively explore the patient’s understanding, concerns, values, and goals related to the patient’s pregnancy and presenting symptoms and provide a differential diagnosis and plan for the patient. The clinical workup is notable for an intrauterine pregnancy with mild subchorionic hemorrhage. The examinee should then summarize the findings on the patient’s labs and imaging, verbalize their concern for a threatened abortion, and provide guidance on next steps and options for care. The examinee should ensure they provide compassionate, empathetic communication — both verbally and nonverbally —ask about the patient’s concerns, values, and goals, and offer support for the patient’s reaction to the information provided. The session will conclude after the examinee clearly states a care plan agreed upon by the patient and examinee (including recommendations, follow-up, and monitoring) and return precautions. They should also allow the patient the opportunity to ask final questions.

The learner enters with the standardized patient seated on the exam table. The patient appears tense with a worried facial expression and is avoiding eye contact. The learner is provided with vital signs for the patient which are stable. The learner is expected to introduce themselves, build rapport, obtain a brief history, provide empathy for the patient who will express concern, and order appropriate diagnostic studies. When the results are available, the learner can request them from the nurse. Prior to discussing with the patient, they should advise the patient that they have the results and ensure they are ready to receive them. When ready, the learner discusses them with the patient. In addition to providing the results, after a moment to allow the patient to absorb the results, the learner should be prepared to answer questions, discuss the diagnosis of miscarriage, and further validate the patient’s concerns if needed. When they deem the patient ready, they should invite the patient to collaborate on their plan of care including pain management if any, follow up, and return precautions.

### Patient-Centered Communication Case: Threatened Miscarriage Standardized Patient Script

#### PATIENT CHARACTERISTICS (Remain consistent throughout case)

- Body Language: Tense, arms crossed or holding abdomen
- Facial Expression: Worried, furrowed brow
- Eye Contact: Avoidant, looks down or away
- Speech: Talkative and questioning early in the encounter; she becomes quieter and more subdued after receiving her ultrasound results

#### TIME STATE 1: Chief Complaint and History

SP Opening Line (begin with this):

“I’ve been bleeding from my vagina and my stomach hurts. It started yesterday.”

If the learner asks the following questions, answer as follows:

- What medicine did you take?
  ○ “I took a Tylenol. It helped a little with the pain, but not much.”
- When was your last period?
  ○ “About seven weeks ago.”
- Any medical problems or allergies?
  ○ “Nope. I don’t have any health problems or allergies.”
- Have you ever been pregnant before? Were you trying to get pregnant?
  ○ “I’ve never been pregnant before. I have a partner and we’ve talked about having kids someday, but we weren’t trying right now.”

SP Cue (Give this line near the end of Time State 1 to prompt empathy):

“I’m really scared something’s wrong.”

SP Cue (Give this line to prompt the learner to discuss the tests ordered for the plan of care):

“What do you think is going on with me? What kinds of tests will you order for me?”

*Transition to Time State 2 only when the learner starts discussing test results.*

#### TIME STATE 2: Test Results and Emotional Response

Nurse colleague states, “the test results are ready.” (*Learners should request the test results to be provided to them*).

SP starts with:

“What do you think is going on with me, doctor?”

Follow-up questions (Use only if the learner does not offer information or to prompt specific responses):

1. “Can you explain the results to me in a way I can understand?” *(Cue for layperson communication)*
2. “I’m afraid of what you are telling me. Are you sure about the results? How do you know?” *(Cue for assessing uncertainty and communication style)*
3. “I feel guilty... did I do something to cause this?” *(Cue for emotional validation and support)*
4. “So what are my options now?” *(Cue for shared decision-making)*

If the learner is too technical or vague (not using simple language or is not direct), SP should appear confused and say,

“I’m not following. Can you say that another way?”

*Transition to Time State 3 when collaborative planning begins with the patient*

#### TIME STATE 3: Future Planning and Reassurance

Learners should transition to form a collaborative plan of care with the SP at this time. The learner should make a statement that asks about the SP’s goals for this pregnancy. If not, the SP will state:

“I really want to keep this pregnancy. What are my options? What should I do now?”

Additional Cues (Space these out to allow the learner to respond):

1. “Am I going to be okay? What happens if the bleeding doesn’t stop?”
2. “Is my baby going to be okay?”
3. “My sister had surgery during her pregnancy and now can’t have kids. Could that happen to me?”

If the learner reassures the SP without a clear follow-up plan, say:

“Okay… but how will I know if something’s wrong again?”

*Transition to Time State 4 when the learner begins to confirm understanding the follow up instructions.*

#### TIME STATE 4: Closing the Encounter

SP only moves here if the learner gives a plan and asks about the SP’s understanding of the plan.

SP will state:

“Got it—I’ll go to the follow-up and come back if anything gets worse.”

If asked, “Do you have any questions?” say, “No, I think you explained it. Thanks.”

INSTRUCTIONS FOR THE SP

- Use plain, emotional language. Do not use medical terms unless the learner introduces them first.
- If the learner seems stuck or quiet for more than 10 seconds, use one of your prompting lines to help them move forward.
- Stay on script — don’t improvise or give information unless asked.
- Use one cue per Time State unless prompted otherwise. Prioritize the first listed cues.

## CERTIFYING EXAM ASSESSMENT

*Patient-Centered Communication Case: Threatened Miscarriage*

Learner: _________________________________________

**Establish rapport**

□ Introduce self as “Doctor”
□ Establish rapport through eye contact, using open body language, and engaging the patient in dialogue.

**Acknowledge and respect the patient’s perspectives**

□ Use open-ended questions to understand their position
□ Ask clarifying questions
□ Elicit key history (bleeding, cramping, last menstrual period, and pregnancy intention)

**Communicate information effectively to facilitate mutual understanding**

□ Be concise
□ Use plain language
□ Express empathy and normalize emotional responses
□ Address guilt without assigning blame.
□ Check with the patient for understanding throughout the encounter and invite the patient to ask questions before ending

**Share results of relevant diagnostic studies and other information**

□ Clearly explain the lab and ultrasound results in layperson terms
□ Acknowledge uncertainty and provide context

**Support shared decision-making to optimize outcomes**

□ Provide options, elicit patient goals regarding pregnancy management
□ Discuss follow-up plan (OB/gyn referral, monitoring)
□ Provide clear return precautions.

**Summative and formative comments:**

### Stimulus Inventory

[Table t5-jetem-10-5-ce58] [Table t6-jetem-10-5-ce58]

[Fig f1-jetem-10-5-ce58]

## References

[1] Benson LS, Holt SK, Gore JL (2023). Early pregnancy loss management in the emergency department vs outpatient setting. JAMA Netw Open.

[2] Hernandez J, Frallicciardi A, Nadir NA, Gothard MD, Ahmed RA (2020). Development of a Simulation Scenario Evaluation Tool (SSET): modified Delphi study. BMJ Simul Technol Enhanc Learn.

[3] Eppich W, Cheng A (2015). Promoting Excellence and Reflective Learning in Simulation (PEARLS): development and rationale for a blended approach to health care simulation debriefing. Simul Healthc.

[4] Bajaj K, Meguerdichian M, Thoma B, Huang S, Eppich W, Cheng A (2018). The PEARLS healthcare debriefing tool. Acad Med.

[5] Gu C, He Y, Li X, Li Q, Xuan Q, Li K (2023). The effects of first-trimester subchorionic hematoma on pregnancy outcomes: a retrospective cohort study. Arch Gynecol Obstet.

[6] Hashim MJ (2017). Patient-Centered Communication: Basic Skills. Am Fam Physician.

[7] Complications of Pregnancy ClinicalKey (Internet).

[8] Heaton HA, Tintinalli JE, Ma OJ, Yealy DM (2020). Ectopic pregnancy and emergencies in the first 20 weeks of pregnancy (Internet). Tintinalli’s Emergency Medicine: A Comprehensive Study Guide.

[9] Resende GP Subchorionic hemorrhage.

